# A 12-Week Exercise Program for Pregnant Women with Obesity to Improve Physical Activity Levels: An Open Randomised Preliminary Study

**DOI:** 10.1371/journal.pone.0137742

**Published:** 2015-09-16

**Authors:** Michèle Bisson, Natalie Alméras, Sébastien S. Dufresne, Julie Robitaille, Caroline Rhéaume, Emmanuel Bujold, Jérôme Frenette, Angelo Tremblay, Isabelle Marc

**Affiliations:** 1 Department of Pediatrics, Centre hospitalier universitaire (CHU) de Québec, Laval University, Québec City, Province of Québec, Canada; 2 Department of Kinesiology, Laval University, Quebec City, Province of Québec, Canada; 3 Department of Rehabilitation, CHU de Québec, Laval University, Québec City, Province of Québec, Canada; 4 Department of Food Science and Nutrition, Laval University, Québec City, Province of Québec, Canada; 5 Department of Family Medicine and Emergency Medicine, Laval University, Québec City, Province of Québec; 6 Department of Obstetrics and Gynecology, CHU de Québec, Laval University, Québec City, Province of Québec, Canada; University of Alabama at Birmingham, UNITED STATES

## Abstract

**Objective:**

To evaluate whether a 12-week supervised exercise program promotes an active lifestyle throughout pregnancy in pregnant women with obesity.

**Methods:**

In this preliminary randomised trial, pregnant women (body mass index ≥ 30 kg/m^2^) were allocated to either standard care or supervised training, from 15 to 27 weeks of gestation. Physical activity was measured by accelerometry at 14, 28 and 36 weeks, while fitness (oxygen consumption (VO_2_) at the anaerobic threshold), nutrition (caloric intake and macronutrients percentage) and anthropometry were assessed at 14 and 28 weeks of gestation. Analyses were performed using repeated measures ANOVA.

**Results:**

A total of fifty (50) women were randomised, 25 in each group. There was no time-group interaction for time spent at moderate and vigorous activity (p_interaction_ = 0.064), but the exercise group’s levels were higher than controls’ at all times (p_group effect_ = 0.014). A significant time-group interaction was found for daily physical activity (p = 0.023); similar at baseline ((22.0 ± 6.7 vs 21.8 ± 7.3) x 10^4^ counts/day) the exercise group had higher levels than the control group following the intervention ((22.8 ± 8.3 vs 19.2 ± 4.5) x 10^4^ counts/day, p = 0.020) and at 36 weeks of gestation ((19.2 ± 1.5 vs 14.9 ± 1.5) x 10^4^ counts/day, p = 0.034). Exercisers also gained less weight than controls during the intervention period despite similar nutritional intakes (difference in weight change = -0.1 kg/week, 95% CI -0.2; -0.02, p = 0.016) and improved cardiorespiratory fitness (difference in fitness change = 8.1%, 95% CI 0.7; 9.5, p = 0.041).

**Conclusions:**

Compared with standard care, a supervised exercise program allows pregnant women with obesity to maintain fitness, limit weight gain and attenuate the decrease in physical activity levels observed in late pregnancy.

**Trial Registration:**

ClinicalTrials.gov NCT01610323

## Introduction

Physical activity during pregnancy can increase cardiorespiratory fitness [1], decrease gestational weight gain [2] and lower the risk of preeclampsia [3]. However, such benefits remain uncertain in women with obesity. These women are spontaneously less active than their lean counterparts [4], which may exacerbate their already low fitness levels [5] and risk of excessive gestational weight gain [6]. Consequently, exercise programs targeting this population are needed, as they can potentially decrease the risk of perinatal complications.

Increasing exercise levels in pregnant women with obesity appears challenging, as adherence to exercise programs has been of concern in previous trials [7, 8]. Moreover, the efficacy of such interventions to improve physical activity levels throughout pregnancy is usually not objectively measured. Although recommendations have been proposed for pregnant women with obesity [9, 10], their impact on maternal fitness have been poorly studied and accordingly, the type, volume and intensity of physical activity required to maintain fitness in this population is unknown.

As face-to-face, individualized physical activity interventions have the potential to improve adherence to an active lifestyle [11], we sought to investigate its effect in pregnant women with obesity. The primary objective of this study was to evaluate whether an individually supervised, 12-wk moderate-intensity exercise program during the 2^nd^ trimester of pregnancy results in higher physical activity levels throughout pregnancy in women with obesity.

## Materials and Methods

### Study design

Recruitment for this randomized controlled parallel-group study with a 1:1 allocation ratio was performed at the Centre Hospitalier Universitaire (CHU) de Québec and the Centre de santé et de services sociaux de la Vieille-Capitale, from October 2011 to November 2013, with follow-ups completed in June 2014. The intervention and fitness tests took place at the *Pavillon de prévention des maladies cardiaques* (PPMC, Institut Universitaire de Cardiologie et Pneumologie de Québec). Research Ethics Board of these institutions approved the study, and all participants provided written informed consent. The protocol of the study was registered in ClinicalTrials.gov, following the enrollment of the first participants (NCT01610323, URL https://clinicaltrials.gov/ct2/show/study/NCT01610323?term=NCT01610323&rank=1). As implementing an exercise intervention can be challenging, initial recruitments for this preliminary study aimed at confirming the intervention feasibility. Registration was delayed until funding allowed us to continue the recruitment for this preliminary study. At the time of registration, only 5 participants had completed the primary outcome assessment, which was originally cardiorespiratory fitness following the intervention as mentioned in the original protocol. However, cardiorespiratory testing in pregnant women with obesity raised feasibility issues, especially at 28 weeks, and accordingly the primary outcome was modified for the impact of the intervention on physical activity levels, in accordance with the registered protocol and the sample size requirement for the physical activity outcome (as describe in the “Sample size” section). There are no ongoing trials for this intervention.

### Participants

Participants were recruited before the end of the 14^th^ week of gestation at family practice, obstetrical and ultrasound clinics and in the community. Women with a pre-pregnancy body mass index (BMI) ≥ 30.0 kg/m^2^, regardless of previous physical activity levels, were eligible if they were 18 years or older, presented a singleton pregnancy and planned to deliver in participating hospitals. Women with diabetes or chronic hypertension before pregnancy were excluded. The absence of physical activity contraindications was verified with the women’s physician and the Physical Activity Readiness Medical Examination for Pregnancy (PARmed-X [12]).

### Randomisation

Following baseline assessment, participants were randomly allocated to either the exercise intervention or usual activity. Randomization was stratified according to parity and based on a computer-generated random numbers table. Sealed envelopes were kept in a secure place by a research assistant not involved in the study and provided to a kinesiologist at the time of allocation. Due to the nature of the intervention, kinesiologists in charge of training and participants were not blinded to group assignment. However, all assessors and research assistants in charge of data entry and analyses were blinded to participants’ allocation (defined as “group 1” and “group 2”).

### Study protocol

At 14 weeks of gestation (Visit 1), participants were assessed for physical activity, anthropometry, fitness and fetal growth. Physical activity prior to pregnancy, socio-demographic characteristics and obstetrical history were collected by a trained research assistant. Food intakes were also documented, but no recommendations were made regarding them.

The same measurements were performed at 28 weeks (Visit 2), following the intervention period. Finally, women were met at 36 weeks (Visit 3) to document physical activity during the third trimester. Within 72h following delivery, newborn’s anthropometry was evaluated by a trained research assistant. Medical charts were reviewed to collect perinatal outcomes and birth weight.

### Study groups

The exercise group was offered a supervised exercise program starting at the 15^th^ week of gestation with free membership in a hospital-based conditioning centre, where kinesiologists were always available for counselling. Participants were individually supervised once a week and invited to complete two more sessions/wk. Consistent with the American College of Sports Medicine Guidelines [10], exercise prescription consisted of 3 weekly 1h sessions, for a total of 36 prescribed sessions over 12 wks.

Each session included a 5–10 min warm-up on a stationary ergocycle, a 15–30 min treadmill walk, a 20 min muscular work-out and a cool-down period. Duration of the cardiovascular training increased progressively from 15 min during the first week to 30 min by the end of the first month. The muscular work-out included dynamic exercises for both lower and upper limbs using the participant’s own body weight, small weights, exercising balls and strength equipment with selective charges. Participants started with 1 set of 10–15 repetitions per exercise and progressed to 2 sets of 15 repetitions, with intensity adjusted to their tolerance level. To enhance motivation, the muscular work-out was modified every 4 weeks (twice during the intervention period). Exercise intensity was self-monitored with heart rate monitors (Polar FT4, Polar Electro, Finland) and the modified Borg Scale [13], with targets at 70% of peak heart rate (measured during the fitness test), and/or at a perceived exertion score of 3-5/10. Participants recorded duration and mean heart rate of each session from their monitors on their exercise log. On non-training days, women were advised to be as active as possible.

The control group was told to continue usual activities without being restrained from doing physical activity. Both groups were given a pamphlet (from Kino-Québec, an agency promoting physical activity) about the benefits of physical activity and appropriate exercises for pregnant women [14].

### Outcomes assessment

Physical activity was measured by accelerometry at 14, 28 and 36 weeks of gestation. Women were instructed to wear the accelerometer (GT3X+, ActiGraph, USA) on the hip for 7 consecutive days, with permission to remove it before bedtime. The primary outcome was defined as the time spent at moderate and vigorous physical activity (MVPA) at 36 weeks of gestation. As the number of days with wear time varied across subjects, reporting activity data per day (instead of per week) was more appropriate. Accordingly, we also reported the number of accelerometry counts/day (reflecting total activity), daily time spent at MVPA in periods ≥10 min (minimum duration required to improve fitness [15]) and daily step counts. MVPA was calculated using the Matthews’ cut point [16], previously used in pregnant women with obesity [17]. Accelerometers were operated according to the manufacturer’s specifications, and analyses were performed using Actilife software. Non-wear time (60 min or more of consecutive zeros [18]) was assessed from accelerometry data, with spurious data removed [19]. Per protocol, if accelerometers were worn for less than 8h daily and for less than 5 days, data were excluded [17] from the main analyses. In addition to this analysis, sensitivity analyses without a minimum wear time requirement were conducted, with and without removal of spurious data [19].

Physical activity in the previous month was also measured at each visit using the Pregnancy Physical Activity Questionnaire (PPAQ) [17, 20] which specifies the type of physical activity performed, adding to data collected through accelerometry [21]. Time spent at each activity was multiplied by its intensity (in Metabolic Equivalent of Task (MET)) [22] and summed to obtain a weekly energy expenditure in METs∙h∙wk^-1^.

Adherence to exercise prescription was calculated as the number of completed sessions during the intervention, as collected in the participants’ log and verified with heart rate monitors’ recordings.

Maternal weight was measured using an electronic scale (InBody 520, Biospace, USA) at each visit. Height was measured at Visit 1, and skinfolds (Harpenden skinfold calliper, Baty, UK) were measured at 14 and 28 weeks of gestation by an experienced exercise physiologist, as described elsewhere [5]. Skinfolds were used to estimate fat percentage using the Jackson and Pollock’s equation for women [23]. Weight gain outcomes were weight gain from 14 to 36 weeks of gestation, and weight gain from 14 to 28 weeks (at the end of the intervention period). To account for the different time period between two weight evaluations, the rate of weekly weight gain was reported (weight gain divided by the number of weeks between the two weight evaluations). Total gestational weight gain (difference between the last weight before delivery and pre-pregnancy weight as reported in the medical charts) was also calculated.

Cardiorespiratory fitness, defined as oxygen uptake at the anaerobic threshold (VO_2_ AT), was assessed at 14 and 28 weeks of gestation by a qualified exercise physiologist during a peak treadmill exercise test with gas exchange analysis (Quark B2, version 8.1a, Cosmed, Italy). A standardized procedure was followed [24], using the modified Bruce ramp protocol [25]. VO_2_ AT was identified by two independent exercise physiologists using the V-slope method [26]. Muscular testing included handgrip strength (Model 78010, Lafayette Instrument Company, USA) and isokinetic strength and endurance of the quadriceps (Biodex System 4, Biodex Medical Systems Inc., USA) following standardized procedures [5, 27–29].

Dietary intakes over the last month were measured at 14 and 28 weeks of gestation using an interviewer-administered food frequency questionnaire [30] with use of food models for estimation of portions.

Fetal growth and uterine arteries mean pulsatility index were evaluated by a certified technician during Doppler studies at 14 and 28 weeks of gestation (Voluson E8 Expert system, GE Healthcare Inc., USA). Neonatal anthropometry included length, head circumference (Models 212 and 416, Seca corp, Germany) and skinfolds (Lange skinfold caliper, Beta Technology, USA). Fat mass and percentage were calculated with a validated equation [31], and birth weight Z-scores adjusted for sex and gestational age were based on Canadian references [32].

### Sample size

Sample size was calculated a priori based on previously published accelerometry data reporting that in the third trimester, women with obesity spent 16 ± 16 min/d doing MVPA in bouts ≥10 min [17]. Based on a t-test, a sample size of 21 participants per group allowed detecting an increase of 14 min/d in the intervention group compared to controls, justified by current recommendations (i.e. 30 min/d [33]), with an 80% power and two-sided alpha level at 0.05. With an estimated 15% of losses, 50 participants were recruited.

### Statistical analyses

Data are presented as means ± standard deviation and percentage for continuous and categorical variables, respectively. Analyses were performed using SAS statistical package 9.4 on an intention-to-treat basis. In order to perform a comprehensive analysis of all physical activity measures over time (at 14, 28 and 36 weeks of gestation), repeated measures ANOVA using a linear mixed model were conducted to compare the effect of group allocation (exercise vs control), time (baseline, 28 weeks, 36 weeks) and their interaction on physical activity levels. If a significant “time-group” interaction was found, comparison over time was made for each group separately, and comparison between groups was made at each individual time. Otherwise, main effects were presented. The covariance structure for repeated measures ANOVA was chosen separately for each outcome. The structure with the lowest Akaike Information Crieterion corrected for finite sample (AICc) amongst several of the most popular structures was chosen. The normality assumption of the residuals of the ANOVA was verified by checking the distribution of scaled residuals obtained by the linear mixed model. Skewness and kurtosis coefficients, as well as histograms and Kolmogorov-Smirnov and Shapiro-Wilk tests, were evaluated and confirmed that the assumption was met. Post hoc tests with the Bonferroni correction were performed to take into account multiple comparisons and keep the familywise error rate at 5%. Sensitivity analyses without a minimum wear time requirement for accelerometry were also conducted, with and without removal of spurious data. As pre-pregnancy physical activity levels could influence the physical activity profile during pregnancy, a sensitivity analysis stratified by pre-pregnancy physical activity level was performed for physical activity outcomes, with women dichotomized as “previously active” or “previously inactive” based on the median value of the pre-pregnancy self-reported energy expenditure spent at sports and exercise. For other outcomes (exploratory analyses for weight gain and neonatal outcomes), groups were compared by Student t test, Wilcoxon rank sum test, χ^2^ or Fisher’s exact test.

## Results

**Fig 1:**
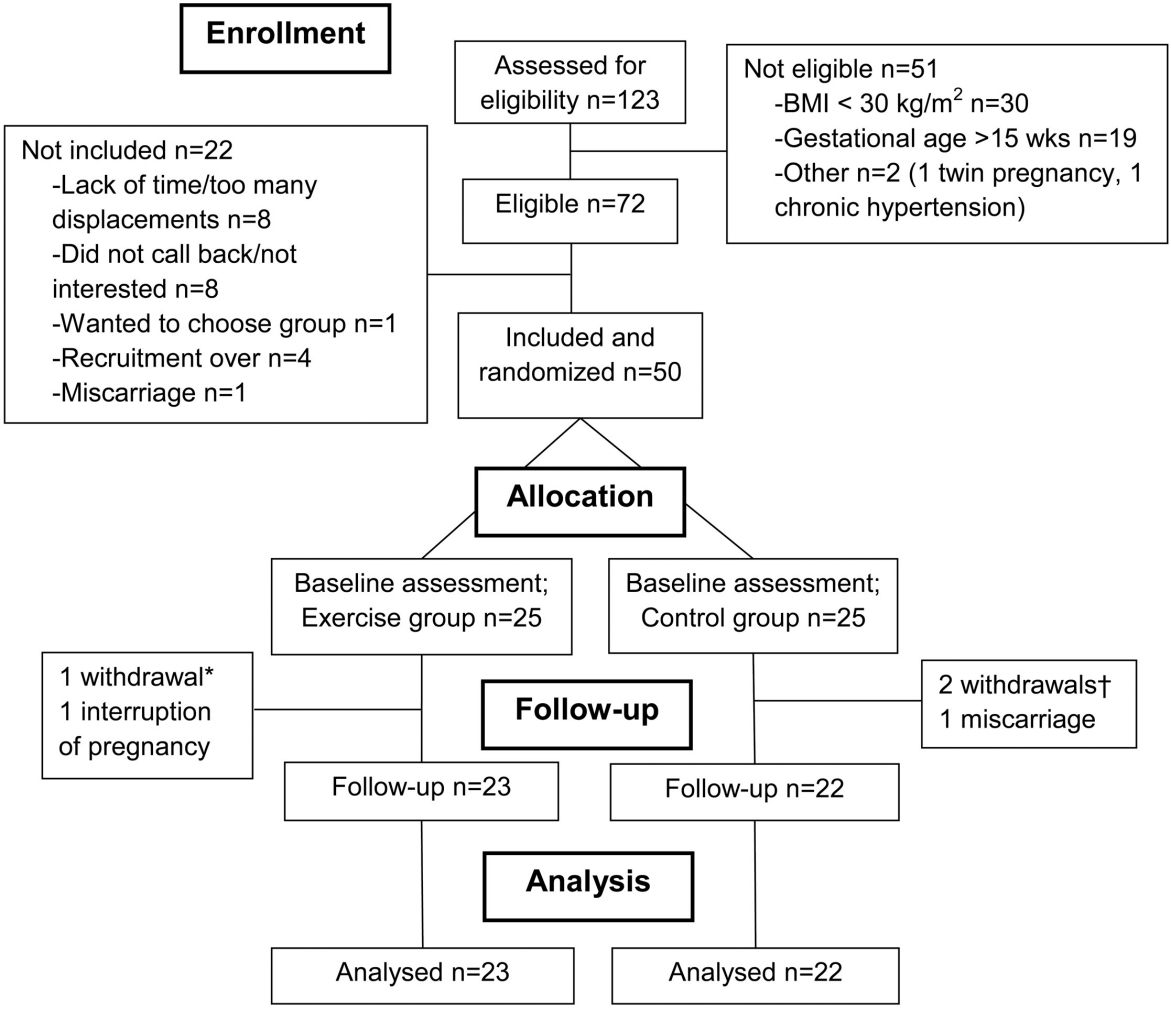
Flowchart. *One participant withdrew after randomization (lack of time); †Two participants withdrew after randomization (unsatisfied with group allocation).

Among 123 interested women, 72 were eligible and 50 were randomized in one of the two study arms (25 per group, see Fig 1). Both groups were similar with respect to baseline socio-demographic characteristics (Table 1). Self-reported physical activity prior to pregnancy was also similar between groups, although the control group reported higher total energy expenditure than the exercise group (Table 1).

**Table 1:** Participants’ characteristics at 14 weeks (Visit 1).

Mean ± SD or *n* (%)	Exercise group n = 25	Control group n = 25	P-value
Age, *year* ^a^	30.5 ± 3.7	31.0 ± 4.0	0.664
White^b^	24 (96)	21 (88)	0.349
Schooling ≥ bachelor degree	15 (60)	16 (64)	0.771
Married or living with a partner	25 (100)	25 (100)	1.000
Employed during 1^st^ trimester	16 (64)	17 (68)	0.765
*Number of work hours/wk*	35.9 ± 8.8	31.0 ± 11.1	0.287
Preventive withdrawal/mandatory leave	9 (36)	8 (32)	0.765
Smoking before pregnancy	1 (4)	2 (8)	1.000
Smoking during pregnancy	1 (4)	0	1.000
Parity ≥ 1	14 (56)	14 (56)	1.000
Gestational age at visit 1, *wk* ^a^	13 ^4/7^ ± 1 ^1/7^	14 ^1/7^ ± 1 ^0/7^	0.053
Pre-pregnancy BMI, *kg/m* ^*2*^	34.6 ± 5.4	33.9 ± 4.5	0.684
Pre-pregnancy BMI by category^b^	-	-	0.232
*Obesity class I (30–34*.*9 kg/m* ^*2*^ *)*	17 (68)	16 (64)	-
*Obesity class II (35–39*.*9 kg/m* ^*2*^ *)*	3 (12)	7 (28)	-
*Obesity class III (≥ 40 kg/m* ^*2*^ *)*	5 (20)	2 (8)	-
Pre-pregnancy weight, *kg*	93.4 ± 17.6	90.7 ± 13.9	0.907
Gestational weight gain at visit 1, *kg* ^a^	1.6 ± 2.1	1.1 ± 3.3	0.539
BMI at visit 1, *kg/m* ^*2*^	35.2 ± 5.4	34.3 ± 4.1	0.877
Total self-reported pre-pregnancy energy expenditure (PPAQ), *METs·h/wk*	243.4 ± 98.1	305.3 ± 174.5	0.036
Energy expenditure by intensity, *METs·h/wk*	-	-	-
*Sedentary*	80.4 ± 26.7	81.1 ± 32.6	0.946
*Light*	83.7 ± 46.3	110.1 ± 58.1	0.099
*Moderate*	72.8 ± 71.2	104.7 ± 117.8	0.076
*Vigorous*	6.5 ± 7.5	9.4 ± 14.3	0.968
Energy expenditure by type, *METs·h/wk*	-	-	-
*Household and care giving*	76.1 ± 54.6	113.6 ± 75.7	0.060
*Occupational activity*	96.3 ± 40.5	104.8 ± 104.3	0.857
*Sports and exercise*	17.8 ± 12.2	18.6 ± 17.6	0.698
*Transportation*	27.0 ± 24.6	27.7 ± 16.5	0.341

^a^Student t-test (other continuous variables evaluated using Wilcoxon rank sum test)

^b^Fisher exact test (other categorical variables evaluated using χ^2^).

### Physical activity assessments

**Fig 2:**
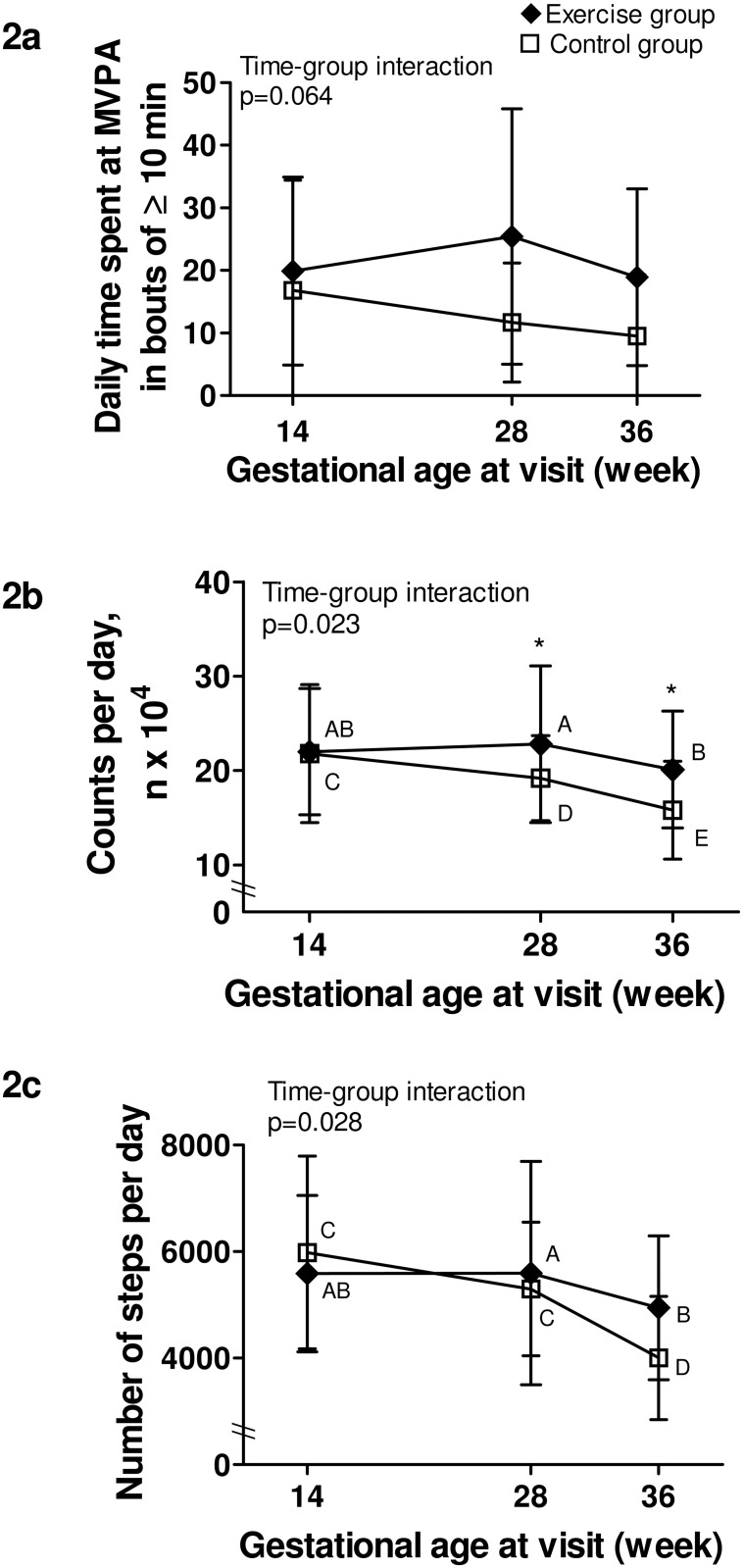
Objectively measured physical activity levels throughout pregnancy. Black lozenge: exercise group. White square: control group. Fig 2a. Daily time spent at moderate and vigorous physical activity in bouts of at least 10 min; Fig 2b. Total activity per day, expressed as the daily number of accelerometry counts; Fig 2c. Number of steps per day. P-value is for time-group interaction significance; * Indicates a significant difference (p<0.05) between groups at a specific time point; Different capital letters (A, B, C, D, E) within a group indicate significant differences between time points.

Physical activity data can be found in Table 2. For daily MVPA in bouts ≥10 min (Fig 2a), there was no significant time-group interaction, although a trend was present. There was a significant group effect (p = 0.014), meaning that the exercise group spent more time doing MVPA in bouts ≥10 min than the control group at all times.

**Table 2:** Physical activity levels throughout the study.

	Baseline at 14 weeks		End of program at 28 weeks		Follow-up at 36 weeks		ANOVA result
Mean ± SD or *n* (%)	Exercise group	Control group	Exercise group	Control group	Exercise group	Control group	P-value for interaction
*Accelerometry*, *n*	23	22	20	17	18	16	-
MVPA in bouts, *min/d*	19.9 ± 15.0	16.8 ± 17.6	25.4 ± 20.4	11.7 ± 9.5	18.9 ± 14.1	9.5 ± 9.8	0.064^a^
Counts per day (n x 10^4^)	22.0 ± 6.7	21.8 ± 7.3	22.8 ± 8.3	19.2 ± 4.5	20.1 ± 6.2	15.8 ± 5.2	0.023
Steps per day	5587 ± 1472	5984 ± 1806	5598 ± 2094	5298 ± 1252	4947 ± 1349	4006 ± 1157	0.028
*Self-reported PA*, *n*	25	25	23	22	23	22	-
Total energy expenditure, *METs·h/wk*	194.6 ± 71.2	226.0 ± 60.0	218.1 ± 67.8	207.8 ± 72.6	185.0 ± 50.8	186.8 ± 83.6	0.070^b^
Energy expenditure by intensity, *METs·h/wk*	-	-	-	-	-	-	-
*Sedentary*	74.4 ± 27.3	79.9 ± 27.3	69.1 ± 28.9	66.4 ± 30.9	62.9 ± 25.3	63.5 ± 25.5	0.65^c^
*Light*	71.8 ± 46.7	86.7 ± 32.5	89.7 ± 41.2	86.3 ± 35.2	73.6 ± 27.7	78.7 ± 43.6	0.23
*Moderate*	46.3 ± 33.7	56.8 ± 37.0	48.5 ± 30.8	54.4 ± 45.1	41.6 ± 25.3	43.8 ± 37.9	0.64
*Vigorous*	2.2 ± 3.4	2.6 ± 8.0	10.7 ± 7.1	0.8 ± 2.0	6.9 ± 5.9	0.8 ± 1.9	<0.0001
Energy expenditure by type, *METs·h/wk*	-	-	-	-	-	-	-
*Household and care giving*	71.7 ± 61.5	91.7 ± 55.4	82.5 ± 52.8	86.7 ± 60.4	75.3 ± 43.4	86.2 ± 76.8	0.39
*Occupational activity*	55.6 ± 41.4	62.3 ± 43.2	62.1 ± 47.3	48.8 ± 55.4	39.7 ± 39.9	27.7 ± 39.1	0.21^d^
*Sports and exercise*	10.2 ± 8.625	8.6 ± 9.9	22.4 ± 13.2	8.4 ± 6.2	15.5 ± 11.2	9.3 ± 7.6	0.002
*Transportation*	21.6 ± 16.9	22.2 ± 12.9	23.3 ± 15.8	25.1 ± 18.4	20.4 ± 12.6	21.0 ± 16.1	0.97

MVPA = moderate and vigorous physical activity; PA = physical activity

^a^significant group effect, p = 0.014; values significantly higher in the exercise vs control group at all time

^b^significant time effect, p = 0.028; values significantly lower at time 3 compared with time 2 in both groups (adjusted p = 0.027)

^c^significant time effect, p = 0.012; values significantly lower at time 3 compared with time 1 in both groups (adjusted p = 0.012)

^d^significant time effect, p = 0.007; values significantly lower at time 3 vs time 1 and time 2 in both groups (adjusted p = 0.001 and p = 0.010).

For total activity reported by the number of counts/day (Fig 2b), there was a significant time-group interaction. Similar at baseline, the exercise group was significantly more active than the control group at both 28 and 36 weeks of gestation (p = 0.020 and p = 0.034, respectively). A significant decline in the number of counts/day between each time point was also found in the control group, whereas the exercise group only decreased their activity levels between 28 and 36 weeks.

A significant time-group interaction was also present for daily step counts (Fig 2c). Although not significantly different between groups at any time, there was a trend towards a higher step counts at 36 weeks in the exercise group compared to controls (p = 0.072). Also, while there was a significant difference in the number of steps per day only between 28 and 36 weeks in the exercise group, the control group showed a step counts at 36 weeks that was significantly lower than those at baseline and at 28 weeks.

**Fig 3:**
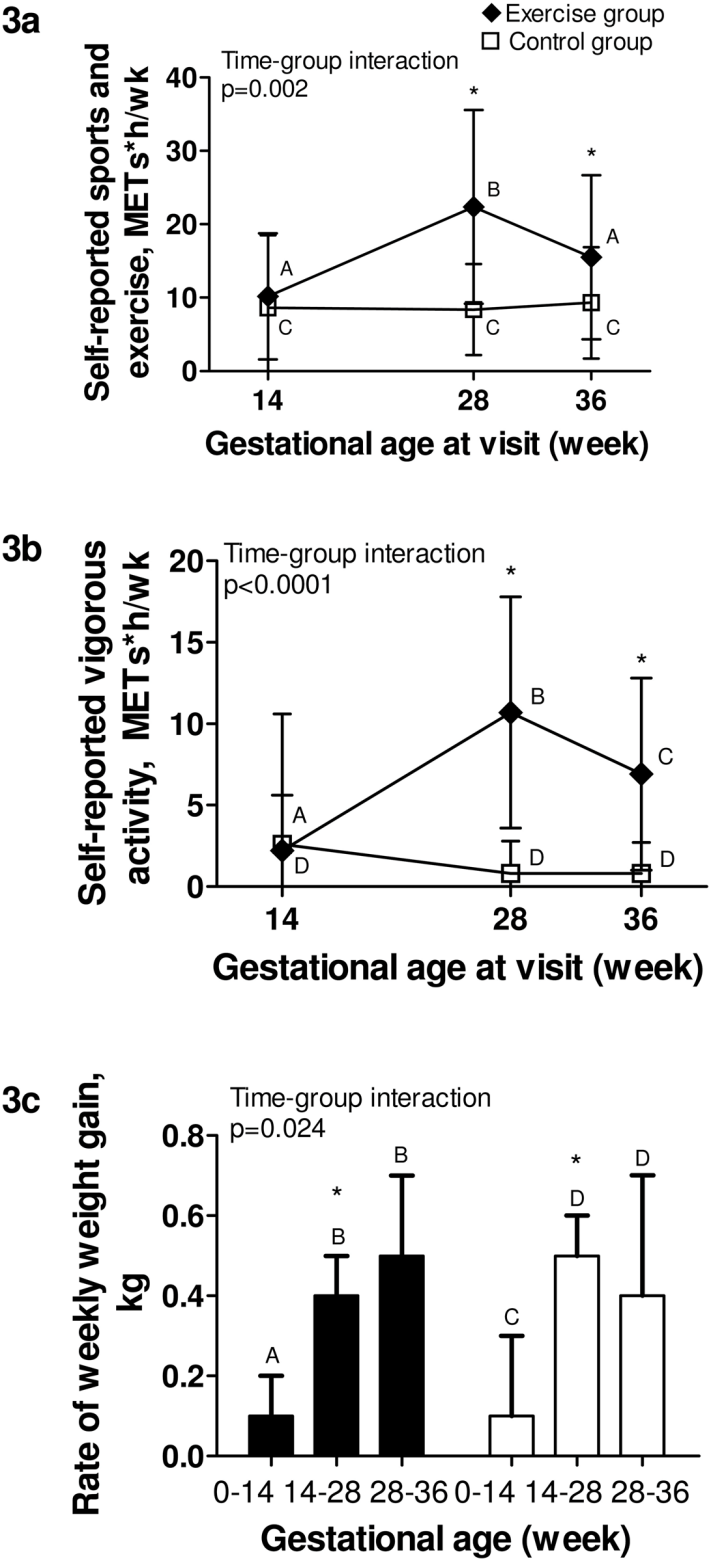
Self-reported physical activity and rate of weekly weight gain throughout pregnancy. Black section: exercise group. White section: control group. Fig 3a. Energy expenditure spent at sports and exercise in the previous month, from the PPAQ; Fig 3b. Energy expenditure spent at vigorous intensity activity in the past month, from the PPAQ; Fig 3c. Rate of weekly gestational weight gain, in kg. P-value is for time-group interaction significance; * Indicates a significant difference (p<0.05) between groups at a specific time point; Different capital letters (A, B, C, D, E) within a group indicate significant differences between time points.

Data from the PPAQ corroborated accelerometry findings (Fig 3a and 3b), as the exercise group reported significantly more time than controls doing sports and exercise activities and vigorous activities at 28 and 36 weeks, respectively. For other domains and intensities of activity from the PPAQ, groups were comparable (Table 2).

Average accelerometer’s daily wear time was 16.1 ± 3.4 h, 15.5 ± 2.8 h and 14.2 ± 1.9 h at 14, 28 and 36 weeks, respectively. Due to drop-outs (n = 5) or insufficient wear time based on our pre-specified requirement (n = 5, 7 and 11 at 14, 28 and 36 weeks, respectively), accelerometry was not available for all participants. However, non-completers’ characteristics were similar in both groups.

Sensitivity analyses without wear time requirement, with and without removal of spurious data, confirmed and even strengthened the results (S1 Table). Moreover, analyses stratified for pre-pregnancy physical activity levels (“previously active” or “previously inactive”) did not suggest significant interactions between pre-pregnancy physical activity levels and physical activity patterns over time in any group (data not shown).

#### Adherence to the intervention

The exercise group performed a total of 18.5 ± 10.1 sessions (1.5 sessions/wk), with 15 (60%) and 5 participants (20%) reaching at least 50% and 75% of the 36 prescribed sessions, respectively. Mean duration was 58.8 ± 4.3 min and exercise heart rate was 121 ± 11 beats·min^-1^ (70.0 ± 5.5% maximal heart rate). There were no adverse events related to the intervention.

### Weight gain

Pre-pregnancy weight and weight gain prior to inclusion in the study did not differ between groups (Table 1). However, the rate of weekly weight gain showed a different profile over time between groups (p-value for interaction = 0.024, Fig 3c). Prior to inclusion in the study, the rate of weekly weight gain did not differ between groups (0.11 ± 0.15 kg/wk vs 0.08 ± 0.23 kg/wk for the exercise and control groups, respectively), while during the program, the exercise group gained less weight per week than the control group (0.35 ± 0.14 kg/wk vs 0.46 ± 0.15 kg/wk for the exercise and control groups, respectively, p = 0.018), despite similar nutritional intakes (Table 3). The control group experienced an increase in fat percentage during this period, as compared to the exercise group (Table 3). However, the rate of weekly weight gain did not differ between groups following the end of the intervention until Visit 3 (0.47 ± 0.24 kg/wk vs 0.45 ± 0.29 kg/wk for the exercise and control groups, respectively), nor did total gestational weight gain for the entire pregnancy (S2 Table).

**Table 3:** Maternal fitness, anthropometry and nutritional intakes at 14 and 28 weeks of gestation.

	Visit 1 (14 weeks)		Visit 2 (28 weeks)		Change from 14 to 28 weeks
Mean ± SD	Exercise group n = 25	Control group n = 25	Exercise group n = 23	Control group n = 22	Difference between groups (95% CI)
VO_2_ AT, *ml∙kg* ^*-1*^ *∙min* ^*-1*^	15.1 ± 2.3^a^	16.0 ± 1.9	15.1 ± 1.6^b^	14.9 ± 2.2	1.1 (-0.03; 1.5)
Change in VO_2_ AT relative to baseline value, *%*	-	-	1.6 ± 13.3^b^	-6.5 ± 9.9	8.1 (0.7; 9.5)^c^
Dominant handgrip strength, *kg*	31.8 ± 4.5	32.3 ± 5.9	31.4 ± 4.7	31.5 ± 5.9	-0.4 (-1.8; 1.0)
Quadriceps strength, *N·m* ^d^	134.6 ± 26.8	140.7 ± 21.6	128.8 ± 33.8	132.2 ± 26.2	3.9 (-13.4; 21.3)
Quadriceps endurance, *N·m* ^d^	706.2 ± 216.4	698.1 ± 125.0	730.0 ± 159.9	717.4 ± 138.2	-10.8 (-111.4; 89.9)
Estimated fat percentage	40.8 ± 6.5	39.8 ± 6.1	40.6 ± 6.6	42.4 ± 4.8	-2.4 (-4.1; -0.8)^e^
Daily caloric intake, *kcal*	2177 ± 724	2198 ± 536	2319 ± 558†	2157 ± 622	122 (-261; 505)
% calories from fat	32.7 ± 4.7	34.9 ± 5.1	31.6 ± 5.3†	33.1 ± 4.2	0.9 (-2.5; 4.4)
% calories from carbohydrates	50.8 ±6.1	49.3 ±5.7	52.1 ± 6.8†	51.3 ± 5.4	-1.0 (-5.2; 3.2)
% calories from proteins	18.8 ± 2.3	17.9 ±2.6	18.6 ± 3.1†	17.7 ± 2.9	-0.1 (-1.7; 1.6)

VO_2_ AT = oxygen consumption at the anaerobic threshold

^a^n = 24

^b^n = 22

^c^p<0.05, Wilcoxon rank sum test

^d^n = 22 and 24 at baseline, and n = 19 and 20 at 28 weeks in exercise and control groups, respectively

^e^p<0.05, Student t test.

### Changes in fitness

At baseline, cardiorespiratory fitness was similar between groups. Following the intervention, VO_2_ AT increased slightly in the exercise group, whereas it decreased in the control group (Table 3). There was no difference between groups for muscular strength and endurance following the intervention (Table 3).

#### Perinatal and neonatal outcomes

At 28 weeks, no differences were found between groups for either mean uterine arteries pulsatility index (data not shown) or estimated fetal weight (1205 ± 169 vs 1219 ± 230 g for exercise and control groups, respectively). There were no differences between groups for birth weight, gestational age at delivery, rate of hypertensive disorders, gestational diabetes or caesarean delivery (S2 Table).

## Discussion

A supervised exercise intervention from 15 to 27 weeks of pregnancy was effective in attenuating the decline in physical activity observed in women with obesity. Indeed, the intervention allowed women to maintain or increase their physical activity levels through the 28^th^ week of pregnancy, whereas it decreased in the control group. This improvement was also supported by a maintained cardiorespiratory fitness level and limited weight gain during the intervention period in the exercise group, compared to controls.

The exercise group also remained more active than the control group during the third trimester, as demonstrated by higher accelerometry counts and self-reported energy expenditure. Despite these higher levels in the exercise group, both groups decreased their activity levels between 28 and 36 weeks of gestation, with values near baseline levels and significantly lower than baseline levels for the exercise and control groups at 36 weeks, respectively. This probably reflects the end of the intervention and the fact that some activities become less comfortable as pregnancy progresses. Therefore, to maintain higher levels of physical activity throughout pregnancy, a follow-up until delivery appears necessary. The advantage of our 12-wk intervention in mid-pregnancy was that it allowed establishing that a supervised exercise program could increase fitness and physical activity levels, with the assessment of a retention effect following the end of the intervention. Seizing the opportunity of pregnancy to promote healthy life habits is important, but taking into account the reality of pregnant women with obesity is also crucial. In that sense, creativity and alternatives to individual, center-based intervention might be needed in late pregnancy to sustain the newly acquired physical activity habit (e.g. follow-up to reinforce behavior, walking club or group activities, or home-based practice).

Increasing physical activity levels with a goal of reaching physical activity recommendations throughout pregnancy is important, as it might help pregnant women in achieving adequate gestational weight gain [34, 35] through an increased energy expenditure, lower their risk of gestational diabetes [35] and fetal macrosomia [36] through a higher muscular glucose uptake [37] and lower their risk of preeclampsia [3] through an anti-inflammatory effect on markers such as C-reactive protein [38] and cytokines [39]. Although the present study was not designed to test these hypotheses, our results remain important as they highlight the feasibility for pregnant women with obesity to as least maintain their physical activity levels during pregnancy and that such levels, even below current recommendations, can induce benefits on cardiorespiratory fitness and gestational weight gain. Indeed, based on the present findings, a combined 1h cardiovascular and muscular moderate-intensity training performed 3 times every two weeks by pregnant women with obesity appears sufficient to maintain fitness and to have a marginal impact on weekly weight gain. Nevertheless, this does not mean that obese pregnant women should stop following current physical activity guidelines; this should be viewed as a minimal threshold to attain in order to reap some health benefits, while more benefits can be expected with higher levels of physical activity [40].

Few studies have focused solely on exercise interventions in pregnant women with obesity, limiting our understanding of the isolated effects of physical activity on maternal and neonatal outcomes. A previous study in pregnant women (BMI ≥ 25 kg/m^2^) did not report significant effects on physical activity levels or weight gain with exercise compared to standard care [7]. However, less than 20% of their participants achieved half the exercise sessions, compared to 60% in the present study. Individual coaching and availability of exercise specialists accustomed to the management of patients with specific needs in the present study might explain these differences. Still, with a goal of 3 exercise sessions/wk, we were expecting women to complete at least 2 sessions/wk. All women were individually supervised once a week, but they had difficulty completing other sessions on their own, suggesting that having an incentive such as a scheduled session with an exercise specialist might be needed to further increase physical activity levels in these women. Other physical activity modalities might facilitate adherence in this population, such as home-based training. Indeed, a recent study showed a 96% adherence to a 6-wk home-based exercise program in diabetic pregnant women [41]. As in the present study, flexible supervision appears as an important component of a successful intervention with pregnant women, either with obesity or high-risk pregnancy.

Although this study focused on physical activity, the absence of nutritional counselling might have reduced the potential for lowering gestational weight gain [42]. The exercise group remained more active than the control group in the 3^rd^ trimester, but the effect on weight gain observed during the intervention did not persist until delivery. It is also important to recognize that even if the intervention had a significant impact on the rate of gestational weight gain during the training period, it was not sufficient to allow women to gain within the Institute of Medicine’s recommended levels for weekly weight gain (0.2–0.3 kg/wk) and for total gestational weight gain (5–9 kg) [43]. However, our single behavior intervention had positive effects on women’s health, without adverse effects on nutritional intakes and no apparent effect on fetal growth. Nevertheless, due to our sample size, conclusions cannot be drawn about the effects of our intervention on weight gain during pregnancy.

Following the 12-wk intervention period, VO_2_ AT decreased by 6.5% in the control group while it increased by 1.6% in the exercise group. This small change in the exercise group could be due to the lower than expected volume of exercise performed by participants (1.5 vs 3 sessions per week), as a dose-response relationship is usually expected between exercise volume and fitness improvement [40]. Indeed, a previous study performed in overweight pregnant women found an 18% increase in VO_2_ AT in their exercise group following a 12-wk intervention [44]. The better adherence found in their study (28 ± 15 sessions over 12 weeks) could partly explain these different findings, as well as the differences in study population characteristics and in the method used to determine the anaerobic threshold. Other potential reasons for the small increase in fitness seen in the present study include the variation in baseline fitness levels between subjects, as those presenting lower levels were probably less active initially, which gave them a better potential for improvement compared to those with a higher fitness level [45], and the interindividual heterogeneity in responsiveness to training (i.e. genetic predispositions) [46]. Nevertheless, although the change over time in VO_2_ AT was relatively small in the exercise group, a training effect was still observed, considering the decreased VO_2_ AT in the control group.

The present study has some limitations. Despite a low drop-out rate (10%), some participants did not adequately complete accelerometry measurements [17], reducing power to show a difference between groups. However, our results remain robust as non-completers were not different between groups and because our results were corroborated by sensitivity analyses and by concordant findings with subjective measures. The social support/interaction with the study staff may have been partially responsible for some observed differences in outcomes between the study arms. However, our trial was pragmatic and objective measurements such as accelerometry and fitness data are less prone to be affected by the support given to participants or by a desirability bias. Fat percentage estimates were based on widely used equations although not validated during pregnancy, as no consensus exists on which anthropometric method should be used to reliably determine body composition during pregnancy [47]. Because it was not possible to have skinfold measures at 36 weeks made by the same assessor as for the first two visits and to avoid high inter-observer variability [48], this assessment was not performed. Finally, results may not be generalizable to all pregnant women with obesity, as our sample included mostly white women with higher education and living with a partner.

## Conclusion

This preliminary study suggests the feasibility of an exercise intervention during pregnancy for women with obesity to enable them to maintain and even increase their physical activity levels, following a supervised exercise program during mid-pregnancy. From a practical perspective, pregnant women with uncomplicated pregnancy should be encouraged to lead an active pregnancy and referred to competent specialists. A minimum of 3 exercise sessions every two weeks appears necessary to maintain fitness in pregnant women with obesity, but a higher volume of exercise might induce greater benefits on other outcomes such as gestational weight gain. Larger trials are needed to determine short and long term benefits of exercise during pregnancy on maternal and child health.

## Supporting Information

S1 CONSORT Checklist Consort Checklist. (DOC)

S1 Protocol Study protocol—French version. (PDF)

S2 Protocol Study protocol—English version. (PDF)

S1 Table Comparison of main accelerometry results using various definitions of non-wear time. (DOCX)

S2 Table Obstetrical and perinatal outcomes. (DOCX)
