# Supplementary material for: A 12-Week Exercise Program for Pregnant Women with Obesity to Improve Physical Activity Levels: An Open Randomised Preliminary Study
Source: PLoS One. 2015 Sep 16;10(9):e0137742. doi: 10.1371/journal.pone.0137742 (PMC4573757; doi:10.1371/journal.pone.0137742)
Supplement: S1 Protocol — (PDF) [file pone.0137742.s002.pdf]

Amélioration de la condition physique chez la femme obèse enceinte : projet d'intervention

Chercheure principale : Isabelle Marc
Co-chercheurs : Natalie Alméras, Caroline Rhéaume, Angelo Tremblay, Emmanuel Bujold
Étudiante : Michèle Bisson, étudiante à la maîtrise en kinésiologie (septembre 2011)

PROTOCOLE

Contexte
L'augmentation de l'obésité chez les femmes en âge de procréer soulève des questionnements quant à son impact sur le fœtus soit directement ou via des troubles métaboliques maternels pendant la grossesse. Des altérations de la tolérance au glucose chez la mère pendant la grossesse, même à des niveaux inférieurs à ceux observés dans le diabète gestationnel, augmentent le risque pour l'enfant (1) d'avoir un poids élevé pour l'âge gestationnel et (2) de présenter des troubles métaboliques au sang de cordon. Ces deux conditions seraient potentiellement associées au développement d'une obésité et/ou à une augmentation des risques métaboliques ultérieurement chez l'enfant. Le manque de conditionnement physique chez les femmes obèses pourrait potentialiser la morbidité de l'obésité sur la tolérance au glucose et sur d'autres indicateurs métaboliques (sensibilité à l'insuline, métabolisme des lipides) pendant la grossesse. Ainsi, l'exercice physique chez la femme obèse enceinte pourrait avoir des bénefices sur la croissance et le statut métabolique du fœtus et potentiellement des effets à long terme chez l'enfant.

Hypothèse
Les femmes obèses qui suivent un programme d'exercice durant le 2ème trimestre de la grossesse amélioreront leur condition cardio-respiratoire et musculaire, leur tolérance au glucose et le risque de troubles métaboliques et inflammatoires. En retour, les risques d'avoir un poids élevé pour l'âge gestationnel et des anomalies métaboliques au sang du cordon seront diminués. Ultimement, une meilleure *condition métabolique* du nouveau-né diminuerait le risque d'obésité dans l'enfance

Objectifs
Déterminer si, par rapport aux soins standards, un programme d'exercice régulier aérobie et musculaire d'intensité modérée au 2e trimestre de grossesse chez la femme obèse enceinte augmente la condition cardio-respiratoire à 28 semaines de gestation.

Devis d'étude
Essai contrôlé randomisé en simple aveugle

Critères d'inclusion: femmes enceintes ≥ 18 ans, grossesse unique, planifie d'accoucher au CHUQ, IMC avant la grossesse ≥ 30,0 kg/m^2. Accord du médecin pour faire de l'activité physique.
Critères d'exclusion: fœtus multiples, diabète ou hypertension chronique avant la grossesse, thyroïde non contrôlée, contre-indication à l'exercice (voir tableau)

Intervention

Dans le groupe exercice, les participantes vont effectuer 3 séances d'exercices hebdomadaires d'une heure chacune incluant un volet aérobie (marche sur tapis roulant et pédalage sur ergocycle) et un volet musculaire pendant 12 semaines, de la 15e à la 27e semaine de gestation. L'intervention sera supervisée par un kinésiologue au Pavillon de Prévention des Maladies Cardiaques (PPMC) à l'Institut Universitaire de cardiologie et pneumologie de Québec). Le groupe contrôle vaquera à ses activités normales sans conseils particuliers.

Randomisation : Les participantes seront réparties aléatoirement dans un des deux groupes, le groupe exercice ou le groupe contrôle (liste de randomisation par ordinateur, enveloppe scellée au centre de recherche du CHUL). Les évaluateurs (tests de fitness et interprétation) seront à l'aveugle du groupe.

Groupe I: groupe exercice

Dans le groupe exercice, des femmes obèses enceintes auront la possibilité de participer à une intervention consistant en 60 minutes d'exercice aérobie à intensité au moins modérée ainsi qu'à un entraînement musculaire, 3 fois par semaine pour 12 semaines (≥150 min./semaine). Cette intervention aura lieu à partir de la 15e semaine de grossesse jusqu'à 27e, durant le jour ainsi qu'en soirée. L'intervention sera supervisée par un kinésiologue certifié et offerte via le PPMC. L'entraînement sera basé sur les recommandations et programmes d'exercice reconnus pour améliorer la condition physique chez les femmes en surpoids, et combinera des exercices aérobies et musculaires. Durant l'entraînement, les participantes seront étroitement supervisées par un kinésiologue dans un ratio de 4-8 participantes par intervenant. L'entraînement aérobie consistera en une marche sur tapis roulant de 25-30 minutes, 3 fois par semaine pour 12 semaines, en débutant avec des séances de 15 minutes. La durée des séances sera augmentée de 4 minutes/semaine jusqu'à un maximum de 30 minutes par session à la fin du 1er mois d'entraînement. Pour accommoder les participantes, il sera possible dans certaines circonstances (douleurs musculo-squelettiques à la marche, problème d'équilibre, etc.) de substituer le tapis roulant par un entraînement sur ergocycle. Après un échauffement progressif d'une durée de 10 minutes, l'activité aérobie sera réalisée à une intensité d'environ 70 % de la fréquence cardiaque maximale au VO_2 pic (mesuré avant la randomisation). Après la 4e semaine d'entraînement, afin d'optimiser la condition cardiorespiratoire, des intervalles à intensité plus élevée (80-85 % de la fréquence cardiaque maximale au VO_2 pic) entrecoupés d'intervalles à intensité réduite (50 % de la fréquence cardiaque maximale au VO_2 pic) seront progressivement introduits dans la séance, selon la tolérance des participantes. Pour assurer la sécurité des participantes, celles-ci devront coter leur perception d'effort via l'échelle de Borg et devront se soumettre au «Test de la parole». La fréquence cardiaque sera monitorée et enregistrée à l'aide d'un cardiofréquencemètre et celle-ci ne devra pas dépasser 150 batt./minutes (en accord avec les recommandations de la SCPE et de la SOGC). Les exercices musculaires solliciteront les membres inférieurs et supérieurs. Pour ce faire, des poids libres, appareils à charge sélective, ballon, etc. seront utilisés. La séance d'exercice musculaire aura une durée d'environ 15 minutes. Les participantes débuteront par une série de 10 répétitions par exercice et augmenteront progressivement jusqu'à un

maximum de 30 répétitions. Le kinésiologue ajustera l'intensité d'exercice en fonction de la dyspnée et de la fréquence cardiaque des participantes. Celles-ci auront été informées de diminuer leur intensité d'effort en cas de dyspnée ou de fatigue sévère (échelle de Borg $\geq$ 7). L'intensité sera également réduite en présence de symptômes tels les signaux d'alarme (Appendice page 2), de l'inconfort dans la poitrine ou dans les jambes ou des étourdissements. L'entraînement sera répertorié par le kinésiologue et par les participantes dans un journal d'activité physique. Un kinésiologue expérimenté et une étudiante graduée (B.sc. Kinésiologue) seront spécialement formés pour s'assurer que l'intervention soit réalisée selon le protocole et pour évaluer la compliance des participantes au programme.

Groupe II: groupe contrôle

Les participantes du groupe contrôle effectueront leurs activités quotidiennes habituelles sans conseils ni indication particulière.

Dans les 2 groupes, les femmes enceintes recevront les soins usuels pour leur grossesse sans restriction. Toutes médications et autres interventions seront décrites au départ et seront monitorées tout au long du processus.

Issue primaire: condition cardio-respiratoire maternelle (VO2 pic) et tolérance à l'effort (durée et charge soutenue) mesurées par un test de tolérance à l'effort sur tapis roulant à 28 semaines.

Issue secondaire : 1-glycémie à jeun à environ 28 semaines; 2- gain de poids durant la période de l'intervention; 3-endurance et force musculaires mesurées à 28 semaines.

Déroulement de l'étude et des mesures

Les femmes enceintes seront recrutées à l'échographie de datation et évaluées avant la randomisation (13-15 semaines de gestation) et après l'intervention (28-30 semaines). Lors des deux visites: (1) les participantes seront pesées, subiront un test de condition cardiorespiratoire (VO2 pic) sur tapis roulant et (2) L'activité physique sera évaluée par un questionnaire et par accélérométrie (avant et après l'intervention ainsi qu'à 34-36 semaines pour évaluer l'adhérence) et (3) une évaluation nutritionnelle par une nutritioniste utilisera un questionnaire de fréquence alimentaire. (4) Le niveau de stress perçu sera évalué par questionnaire. (5) Une échographie et doppler seront effectuées au baseline et à 32 semaines. Le dossier de la femme sera consulté afin de recueillir les données concernant la grossesse et les complications éventuelles. Le poids à la naissance, la taille et le périmètre crânien seront évalués lors de l'accouchement.

Mesures

1- Évaluation de la condition cardio-respiratoire : Un test d'effort sur tapis roulant sera effectué afin de mesurer la condition cardio-respiratoire des femmes. Les participantes seront également évaluées pour leur condition physique musculo-squelettique. La femme aura eu l'approbation de son médecin pour participer au projet et le test sera effectué sous supervision médicale. Le ParMed-X pour la grossesse sera complété pour s'assurer qu'aucun élément nouveau n'est survenu qui pourrait contre-indiquer l'exercice depuis l'accord du médecin. Le protocole d'effort utilisé sera le

protocole de Balke modifié et publié par Mottola et al [9], et considéré sécuritaire chez la femme enceinte. Le test sera effectué 2 à 3 heures après le dernier repas. Avant le test d'effort, des mesures de repos seront effectuées, soit après 15 minutes de repos en position assise (consommation d'oxygène, tension artérielle, fréquence cardiaque, etc.). Le test d'effort sur tapis roulant débutera avec un échauffement de 5 minutes à une vitesse de 3 mph sur une pente nulle (équivalent à marcher normalement). Toutes les 2 minutes, la pente sera augmentée de 2 %, à vitesse constante. À chaque fin de palier, on demandera à la femme de quantifier sa fatigue de 0 à 10 sur l'échelle de Borg (10 étant le maximum de fatigue). Une fois l'atteinte d'une pente de 12 %, si la fatigue n'est pas survenue, la vitesse sera augmentée par palier de 0,2 mph toutes les 2 minutes. À n'importe quel moment du test, la femme sera autorisée à se tenir légèrement aux rampes de chaque côté du tapis roulant. Le test prendra fin lorsque la femme ressentira une fatigue correspondant à un score de 9-10 sur l'échelle de Borg, ou à n'importe quel moment si elle en ressent le besoin. Une période de 5 minutes de marche normale complétera la procédure pour la récupération progressive. Les paramètres cardio-respiratoires seront enregistrés tout au long du test (fréquence cardiaque, tension artérielle, charge de travail, consommation d'oxygène). Pour des questions de sécurité, la mesure de la condition physique cardio-respiratoire sera déterminée par le VO_2 peak sur la moyenne des 30 dernières secondes. Nous mesurerons également le niveau de vitesse atteinte et la durée de l'exercice. Une collation sera proposée aux participantes après le test d'effort.

2- Évaluation de la condition physique musculo-squelettique : La condition musculo-squelettique sera évaluée avant le test à l'effort à partir du test de force de préhension ainsi que par des procédures standardisées[i,ii,iii] mesurant la force et l'endurance des membres supérieurs et inférieurs à l'aide d'un dynamomètre isocinétique Biodex. Trois aspects importants seront pris en considération dans l'exécution des évaluations dû à la condition de la femme enceinte et aux effets indésirables potentiels : 1) la manœuvre de Valsalva sera évitée, 2) les exercices ne seront pas faits en décubitus dorsal à partir du 2e trimestre et 3) les précautions seront prises pour éviter une hyperextension des articulations due à l'hyperlaxité pendant la grossesse.

La force de préhension sera mesurée à l'aide d'un dynamomètre. La participante tient la poignée du dynamomètre dans le prolongement du bras, à la hauteur de la cuisse et éloignée du corps. Elle serre vigoureusement la poignée en exerçant le maximum de force et en effectuant une expiration pour éviter une hausse de la pression intrathoracique.

Les mesures effectuées au niveau du membre inférieur consistent en des tests de force de 3 répétitions et d'endurance de 15 répétitions réalisés à 60 degrés/seconde au niveau des fléchisseurs et des extenseurs du genou du membre dominant. Ces tests sont effectués en position assise, l'axe de rotation anatomique du genou étant aligné avec l'axe de rotation du dynamomètre, et sont précédés par un échauffement de 5 répétitions à 60 degrés/seconde en incluant une contraction maximale volontaire.

Les mesures effectuées au niveau du membre supérieur consistent en des tests de force de 3 répétitions et d'endurance de 15 répétitions réalisés à 60 degrés/seconde au niveau des fléchisseurs et des extenseurs du coude du membre dominant. Ces tests sont effectués en position assise, l'axe de rotation anatomique du coude étant aligné avec l'axe de rotation

du dynamomètre, et sont précédés par un échauffement de 5 répétitions à 60 degrés/seconde en incluant une contraction maximale volontaire.

3- Niveau d'activité physique : Le niveau d'activité physique avant la grossesse et au 1er trimestre sera évalué au moyen d'un questionnaire semi-quantitatif (Pregnancy Physical Activity Questionnaire, PPAQ) validé chez la femme enceinte et qui prend en considération le mode, la fréquence, la durée et l'intensité de l'activité physique dans plusieurs domaines (récréationnel, occupationnel, le soin des enfants et des adultes, les activités intérieures et extérieures). L'analyse du PPAQ permet de quantifier l'activité globale et son intensité ainsi que de calculer l'énergie moyenne sur une semaine dépensée dans chaque domaine d'activité ou par niveau d'activité. Une quantification objective des niveaux d'activité physique chez les femmes sera mesurée par accélérométrie. Ainsi, après la visite, la femme retournera à la maison avec un accéléromètre et un journal d'activité simplifié. L'analyse des données de l'accéléromètre sur 7 jours permettra de calculer entre autre le nombre de compte total mais aussi le temps passé en activité modérée ou vigoureuse (en comparaison avec des seuils préétablis)

4- Mesures anthropométriques : Les mesures du poids, de la taille et des plis sous-cutanés seront effectuées selon les méthodes standardisées. Le poids sera mesuré à l'aide d'une balance de bioimpédance InBody 520. La mesure de la taille sera effectuée à l'aide d'un stadiomètre avec une précision de 1 mm. Les plis cutanés seront mesurés à l'aide d'un adiposomètre Harpenden de 80 mm avec une précision de 0.2 mm.

5- Alimentation : Les participantes rencontreront une nutritionniste pour une évaluation de leur alimentation au moyen d'un questionnaire de fréquence alimentaire (FFQ).

6- Échographie : la fonction placentaire et le volume placentaire seront évaluées par un doppler des artères utérines en plus de l'échographie de datation avant l'intervention. Les effets de l'exercice sur la croissance fœtale et l'adiposité (échographie 3-D) et sur la fonction placentaire (Doppler de l'artère utérine et ombilicale) seront mesurés en fin d'intervention (32 semaines).

7- Évaluations complémentaires : D'autres variables seront documentées, telles que l'IMC de la mère, le gain de poids depuis le début de la grossesse et durant l'intervention, les données obstétricales (âge gestationnel, parité), le niveau d'éducation, l'ethnicité, les données sociodémographiques et le statut au travail. Les femmes seront interrogées sur leurs habitudes de vie pendant et avant la grossesse (alcool, tabac, drogues illicites, médicaments, etc.). Un questionnaire évaluera la qualité de leur sommeil (questionnaire de Pittsburgh). Enfin, un questionnaire de qualité de vie, le SF36, permettra d'obtenir des données générales sur la qualité de vie des femmes. De plus, le questionnaire PSS-10 permettra d'évaluer le niveau de stress perçu par les femmes et un questionnaire portant sur les barrières à l'adhérence à un mode de vie actif sera rempli par celles-ci à 34-36 semaines. Les mesures de suivi de grossesse (poids, complications, diabète gestationnel, hypertension artérielle de grossesse), de l'accouchement (césarienne) et de la naissance (sexe, poids du bébé, taille, périmètre crânien, hypoglycémie néonatale, apgar) seront recueillies dans le dossier médical de la mère et du bébé. À la naissance, les plis cutanés du bébé et la circonférence du bras seront mesurés.

Taille d'échantillon

Sur la base de SD = 3.14 $ml*min^{-1}*Kg^{-1}$ pour le VO2 pic chez les femmes enceintes à partir des résultats d'une étude antérieure (compatible avec l'étude de Santos chez les femmes en surpoids), un total de 18 participantes par groupe fournira une puissance de 80% pour détecter une différence de 3 $ml*min^{-1}*Kg^{-1}$ ou plus au niveau du VO2 pic avec un alpha = 0,05. Ceci serait en accord avec une publication de Santos et *al.*[iv], qui a démontré une amélioration moyenne de 2,35 $ml*min^{-1}*Kg^{-1}$ suite à un protocole d'exercice similaire au nôtre à 50-60% de la fréquence cardiaque maximale estimée chez des femmes en surpoids enceintes. Nous prévoyons que 15% des données ne seront pas disponibles pour l'analyse de l'issue primaire (VO_2 pic après l'intervention). Nous prévoyons donc recruter 22 participantes par groupe.
Nous estimons que 1 an devrait être nécessaire pour recruter l'ensemble des participantes. Il est à noter que cette étude est aussi une étude de faisabilité.

Risques potentiels pour la sécurité des participantes

La société des obstétriciens et gynécologues du Canada (SOGC) recommande que toutes les femmes sans contre-indication (Appendice) soient encouragées à participer à des exercices aérobies et d'endurance pendant la grossesse[98]. En 2002, *l'American College of Obstetricians and Gynecologists* (ACOG) recommande aux femmes enceintes d'effectuer 30 minutes d'exercice d'intensité modérée par jour, presque tous les jours sinon tous les jours, en absence de contre-indication[46]. Il est recommandé que les femmes inactives soient évaluées avant de débuter un programme d'exercice. En 2008, suivant les recommandations de l'ACOG, le gouvernement américain recommande aux femmes enceintes d'atteindre ≥150 minutes d'activité aérobie modérée pendant la grossesse si elles ne sont pas déjà hautement actives ou si elles ne faisaient pas d'activité d'intensité vigoureuse[99]. Santé Canada endosse ces recommandations et mentionne que l'activité physique devrait être augmentée de façon progressive si la femme entame un programme d'exercice physique au moment de la grossesse[100].

Notre intervention va suivre ces recommandations :

- Absence de contre-indication à l'exercice tel qu'évalué par le médecin traitant : l'approbation du médecin traitant pour la participation de sa patiente au projet de recherche sera obtenue par fax.
- Complétion du PARmed-X pour la grossesse avant le début du programme et réévaluation à chaque séance des signaux d'alarme.
- Tests de condition physique sous supervision médicale
- Recommandations de l'exercice fondées sur les résultats des tests de condition physique et progression dans l'intensité de l'exercice
- Programme en milieu supervisé par un kinésiologue. Un médecin sera disponible si besoin pendant les sessions
- Surveillance par un comité indépendant des risques (prématurité, diabète, pré-éclampsie, saignement, douleurs, problème orthopédiques, poids du bébé).

---

[i] Harbo, T. et *al*., Maximal isokinetic and isometric muscle strength of major muscle groups related to age, body mass, height, and sex in 178 healthy subjects, *Eur J Appl Physiol* Original Article, 2011.

[ii] Danneskiold-Samsøe, B. et al., Isokinetic and isometric muscle strength in a healthy population with special reference to age and gender, *Acta Physiol*, 197 (Suppl. 673), 2009, 1-68.

[iii] Hulens, M. et *al*., Study of differences in peripheral muscle strength of lean versus obese women: an allometric approach, *International Journal of Obesity*, 25, 2001, 676-681.

[iv] Santos, I. et *al*., Aerobic Exercise and Submaximal Functional Capacity in Overweight Pregnant Women: A Randomized Trial, *OBSTETRICS & GYNECOLOGY*, VOL. 106, NO. 2, AUGUST 2005, 243-249.
