# Supplementary material for: A 12-Week Exercise Program for Pregnant Women with Obesity to Improve Physical Activity Levels: An Open Randomised Preliminary Study
Source: PLoS One. 2015 Sep 16;10(9):e0137742. doi: 10.1371/journal.pone.0137742 (PMC4573757; doi:10.1371/journal.pone.0137742)
Supplement: S2 Protocol — (PDF) [file pone.0137742.s003.pdf]

Protocol

Fitness improvement in pregnant women with obesity: intervention study

Context

In pregnancy, the adoption or pursuit of a sedentary lifestyle contributes to the development of co-morbid conditions such as hypertension, maternal and childhood obesity, gestational diabetes, pre-eclampsia, caesarean section and delivery of large-for-gestational-age infants (LGA). Moreover, pregnant women with obesity are spontaneously less active than their lean counterparts, and this may exacerbate their already low fitness levels and risk of adverse pregnancy outcomes. Consequently, exercise programs targeting this population are needed, as they can potentially decrease the risk of perinatal complications.

Hypothesis

Pregnant women with obesity allocated to a supervised, moderate intensity physical conditioning program during the 2nd trimester of pregnancy will improve cardiorespiratory fitness, as compared to women in the control group.

Objective

To evaluate whether a 12-week exercise program in pregnant women with obesity will improve cardiorespiratory fitness following the intervention, as compared to standard care.

Study design

Randomised controlled trial

Inclusion criteria

Pregnant women 18 years or older; single pregnancy; delivery at Centre Hospitalier Universitaire de Québec; pre-pregnancy BMI ≥30 kg/m^2; physician's approval to perform physical activity.

Exclusion criteria

Multiple pregnancy; diabetes or chronic hypertension prior to pregnancy; uncontrolled thyroid problems; exercise contraindications

Study arms

Exercise group: In the intervention group, pregnant women with obesity will be offered participation in an intervention consisting of 60 min of moderate-intensity aerobic and resistance exercise 3 times/week for 12 weeks (≥150 min/week). The intervention will be delivered from 15 to 27 weeks of gestation during days and evenings. It will be supervised by a certified kinesiologist and offered through a specialised conditioning centre in a hospital-based setting. Training will be based on recommendations and will combine aerobic and strength exercises. During training, patients will be closely supervised by a kinesiologist. Aerobic training will consist of treadmill exercise for 25-30 min, starting with 15-min duration/session. The duration will be increased by 4 min/wk up to a maximum of 30 min/session at the end of the 1st month. After a 10-min warm-up,

aerobic activity will be performed at 70% of maximum heart rate at VO2 peak (measured before randomization). Heart rate will be monitored and recorded with a heart rate monitor. Strength exercises will involve the upper and lower limbs. The strength exercise session will last 15 min. Participants will start with 1 set of 10 repetitions per exercise for a maximum of 30 repetitions. The therapist will adjust training intensity according to dyspnea level and heart rate. Participants will be instructed to reduce the intensity of training in case of dyspnea or fatigue (Borg scale >7/10). Training will also be modified or stopped in the presence of adverse symptoms, chest or leg discomfort or dizziness. Training will be recorded by the kinesiologist and by participants in a diary.

Control group: Standard Care, without physical activity restrictions.

Evaluations

Pregnant women will be evaluated before randomisation (at 13-15 weeks) and following the intervention (at 28-30 weeks) for: anthropometric measures, cardiorespiratory fitness (VO_2peak test on treadmill), physical activity levels (by accelerometry and questionnaire at both visits and also at 34-36 weeks to evaluate retention), nutrition, life habits and Doppler study. Medical charts will be reviewed following delivery to collect obstetrical and perinatal data. Anthropometric measures of the newborn will be performed shortly after delivery.

Outcomes

Primary outcome: cardiorespiratory fitness following the intervention
Secondary outcomes: weight gain, muscular strength and endurance, physical activity levels (accelerometry and questionnaire), maternal glycaemia, nutrition, Doppler studies of pulsatility index and fetal growth, sleep quality, quality of life, pregnancy outcomes (birth weight and anthropometric measures, delivery type, pregnancy complications).

Sample size

For this preliminary study, to show a 3 ml/kg/min difference in cardiorespiratory fitness between groups following the intervention, 18 participants per group are required to have an 80% power with an alpha level at 0.05. With an estimated 15% of unavailable data for the primary outcome, a sample size of 22 participants per group will be recruited.

Safety issues

Pregnant women without medical contra-indications are encouraged to lead an active pregnancy. To ensure the safety of the participants, our intervention will follow the recommended guidelines for physical activity during pregnancy (Joint SOGC/CSEP clinical practice guideline: exercise in pregnancy and the postpartum period, 2003). ParMed-X for pregnant women will be reviewed before inclusion, at the cardiorespiratory testing and during the intervention period.
