# Supplementary material for: A 12-Week Exercise Program for Pregnant Women with Obesity to Improve Physical Activity Levels: An Open Randomised Preliminary Study
Source: PLoS One. 2015 Sep 16;10(9):e0137742. doi: 10.1371/journal.pone.0137742 (PMC4573757; doi:10.1371/journal.pone.0137742)
Supplement: S1 Table — (DOCX) [file pone.0137742.s004.docx]

**Supplementary Table 1. Comparison of main accelerometry results using various definitions of non-wear time.**

| Mean ± SD or *n* (%) | Baseline at 14 weeks | | End of program at 28 weeks | | Follow-up at 36 weeks | | P-value for interaction (ANOVA) |
| --- | --- | --- | --- | --- | --- | --- | --- |
|  | Exercise group | Control group | Exercise  group | Control  group | Exercise group | Control  group |  |
| *Spurious data removed, minimum wear time, n* | 23 | 22 | 20 | 17 | 18 | 16 | **-** |
| MVPA in bouts, *min/d* | 19.9 ± 15.0 | 16.8 ± 17.6 | 25.4 ± 20.4 | 11.7 ± 9.5 | 18.9 ± 14.1 | 9.5 ± 9.8 | 0.064 |
| Counts per day (n x 10^4^) | 22.0 ± 6.7 | 21.8 ± 7.3 | 22.8 ± 8.3 | 19.2 ± 4.5 | 20.1 ± 6.2 | 15.8 ± 5.2 | 0.023 |
| *Spurious data conserved no minimum wear time, n* | 25 | 25 | 23 | 22 | 23 | 22 | - |
| MVPA in bouts, *min/d* | 17.7 ± 14.7 | 16.4 ± 17.5 | 22.8 ± 19.9 | 9.3 ± 7.1 | 15.3 ± 13.6 | 6.6 ± 7.4 | 0.033 |
| Counts per day (n x 10^4^) | 20.8 ± 7.0 | 20.6 ±7.6 | 21.4 ± 8.7 | 16.4 ± 4.5 | 17.2 ± 7.5 | 12.3 ± 6.1 | 0.002 |
| *Spurious data removed, no minimum wear time, n* | 25 | 25 | 23 | 22 | 23 | 22 | - |
| MVPA in bouts, *min/d* | 17.7 ± 14.7 | 16.6 ± 17.8 | 23.1 ± 19.8 | 9.6 ± 7.7 | 15.3 ± 13.6 | 6.9 ± 7.9 | 0.029 |
| Counts per day (n x 10^4^) | 20.7 ± 7.0 | 20.6 ± 7.6 | 21.5 ± 8.4 | 16.6 ± 4.6 | 17.2 ± 7.4 | 12.5 ± 6.4 | 0.002 |

Spurious data = 1 minute of isolated activity surrounded by consecutive zeros (inactivity for at least a 60 minutes period); Minimum wear time = at least 5 days with at least 8h of recordings; MVPA = moderate and vigorous physical activity; P-value is for time-group interaction in repeated measures ANOVA.
