# Supplementary material for: A 12-Week Exercise Program for Pregnant Women with Obesity to Improve Physical Activity Levels: An Open Randomised Preliminary Study
Source: PLoS One. 2015 Sep 16;10(9):e0137742. doi: 10.1371/journal.pone.0137742 (PMC4573757; doi:10.1371/journal.pone.0137742)
Supplement: S2 Table — (DOCX) [file pone.0137742.s005.docx]

**Supplementary Table 2. Obstetrical and perinatal outcomes.**

| Mean ± SD or *n* (%) | Exercise group  n=24 | Control group  n=24 | Difference between groups or OR (95% CI) |
| --- | --- | --- | --- |
| Total gestational weight gain, *kg* | 12.3 ± 4.0 | 12.2 ± 5.9 | 0.03 (-2.9; 3.0) |
| Excessive gestational weight gain^a^ | 21 (88) | 17 (71) | 1.24 (0.92; 1.66) |
| Gestational age at delivery, *wk* | 39 ^4/7^ ± 1 ^1/7^ | 39 ^2/7^ ± 1 ^2/7^ | 0 ^2/7^ (-0 ^3/7^; 0 ^6/7^) |
| Caesarian delivery | 8 (33) | 8 (33) | - |
| Gestational diabetes | 3 (13) | 5 (21) | 0.60 (0.16; 2.23) |
| Gestational hypertension^b^ | 2 (8) | 3 (13) | 0.67 (0.12; 3.64) |
| *Newborn outcomes* | - | - | - |
| Birth weight, *g* | 3575 ± 425 | 3455 ± 368 | 121 (-111; 352) |
| Placental weight, *g*^c^ | 541 ± 125 | 542 ± 92 | -1 (-66; 64) |
| Male infant | 10 (42) | 14 (58) | - |
| Birth weight Z-score | 0.325 ± 0.908 | 0.137 ± 0.904 | 0.187 (-0.339; 0.714) |
| Large for gestational age | 4 (17) | 3 (13) | 1.33 (0.33; 5.33) |
| Small for gestational age | 0 | 2 (8) | - |
| Birth length, *cm* | 50.2 ± 2.0 | 49.4 ± 3.1 | 0.8 (-0.7; 2.3) |
| Head circumference, *cm* | 35.0 ± 1.2 | 34.9 ± 1.2 | 0.03 (-0.7; 0.7) |
| Estimated fat mass, *g*^d^ | 602 ± 148 | 564 ± 181 | 39 (-64; 141) |
| Fat percentage^d^ | 16.7 ± 2.8 | 16.2 ± 4.1 | 0.5 (-1.6; 2.7) |

^a^Based on the Institute of Medicine Recommendations; ^b^No participant developed pre-eclampsia; ^c^n=23 in both groups; ^d^n=23 and 19 for exercise and control groups, respectively
